# LLMs displaying less cognitive bias are not necessarily better decision makers

**DOI:** 10.1038/s42256-026-01208-w

**Published:** 2026-03-17

**Authors:** Vittoria Dentella, Marco Marelli, Luca Rinaldi

**Affiliations:** 1 Department of Brain and Behavioral Sciences, University of Pavia, 27100 Pavia, Italy; 2 Department of Psychology, University of Milano-Bicocca, 20126 Milano, Italy; 3 NeuroMI, Milan Center for Neuroscience, 20126 Milano, Italy; 4 Cognitive Psychology Unit, IRCCS Mondino Foundation, 27100 Pavia, Italy

## Introduction

As Large Language Models (LLMs) are increasingly integrated into our lives, the scientific community has a crucial role in illustrating their properties, exploring their controversial sides, and shaping the impacts they might have on aspects of society, including –among others– our cognition [1]. LLMs generate language, and language is a powerful tool for learning. As the usage of LLMs becomes ubiquitous, the texts they produce propagate and progressively form the bedrock for the transmission of knowledge: it is thus essential to be fully aware of the type of knowledge that is transmitted.

At a basic level, LLMs are affected by what is most frequent in their training data. More precisely, LLMs identify, learn and reproduce patterns of word usage: as their primary objective is that of optimally predicting words, LLMs tend to replicate those usage patterns that are most recurrent. The natural consequence of this modus operandi is the amplification of any biases that are encoded in a word’s distributional history [2]. Broadly speaking, these biases can be categorized into two types: social, and cognitive.

**Fig. 1:**
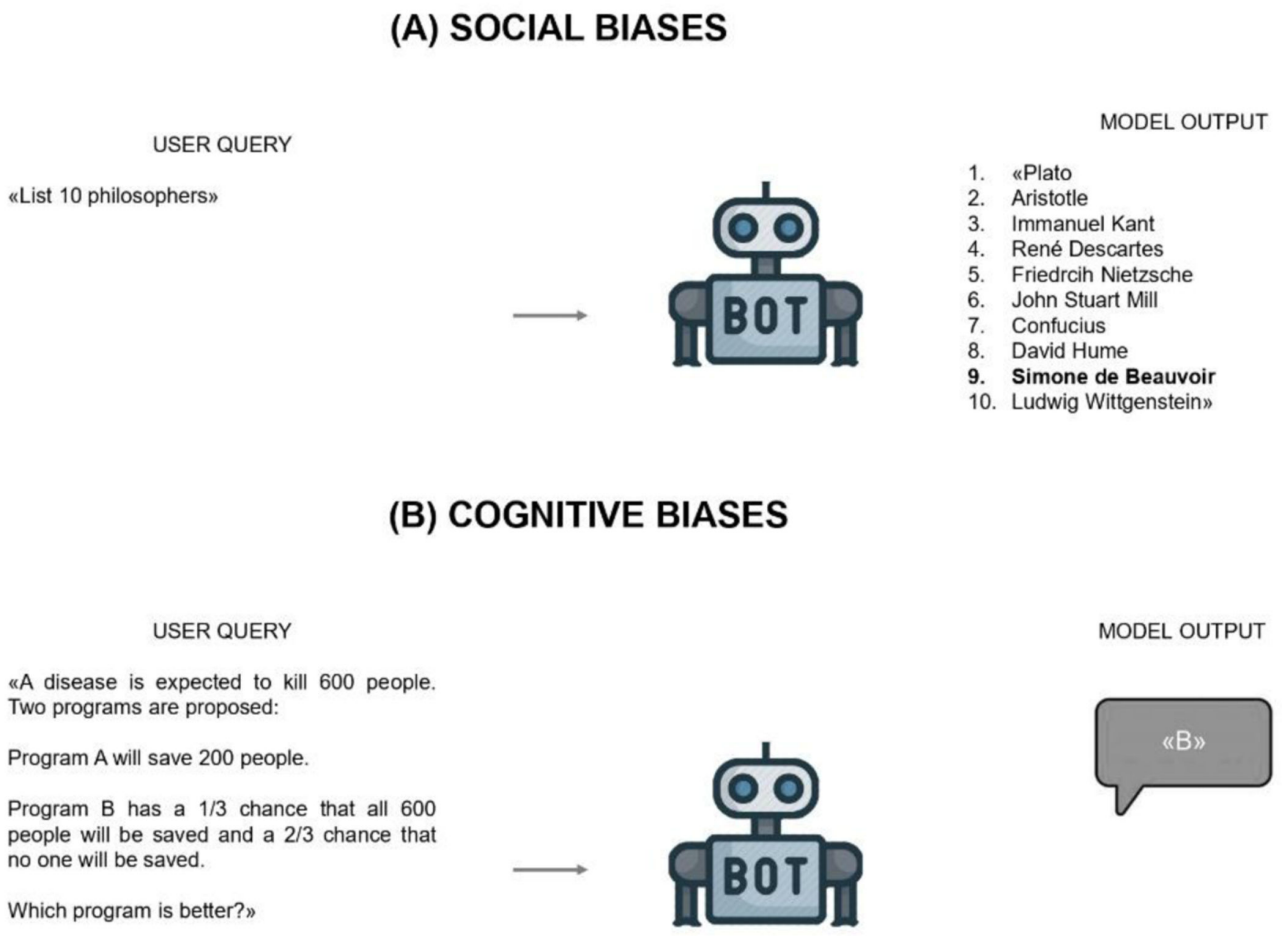
Examples of a social and a cognitive bias. In (A), Simone de Beauvoir is the only female philosopher in the list. In (B), the second treatment is preferred to the first one, despite the two having identical outcomes.

Social stereotypes are culturally derived attitudes towards societal aspects of reality. Gender, ethnic, class and racial stereotypes all fall within this category, and all are picked up by LLMs. Concretely, a social stereotype is found when, given two sets of words differing along a certain dimension (e.g., gender, as for female vs. male names), one of the two sets is more closely associated to one of two sets of attributes, such as family- vs. career-oriented vocabulary. This connection need not be textually explicit: to be prejudiced, corpora do not need to overtly speak of women doing housework as opposed to men performing intellectual jobs. For a LLM to pick up these stereotypes, in fact, it suffices for them to be implicitly encoded in a word’s co-occurrence statistics – and it is precisely these statistics that determine LLMs’ outputs [2]: if male names are more closely associated with the word philosopher, then LLMs are likely to describe a male philosopher when prompted for a description [Fig. 1A]. There is broad consensus on the need to remove stereotyped social views from training data and downstream AI applications, as their perpetuation obstructs efforts to develop fairer attitudes towards historically marginalized groups [3]. Consequently, to limit their capacity as amplifiers of societal inequalities, considerable effort is being put into the characterization and debiasing of LLMs’ representations [4].

Cognitive biases, instead, are systematic departures from normative reasoning, often heuristics-based, which are deeply ingrained in human cognizing: cognitive biases indeed affect perception, reasoning, and processes of memory, learning, and attention, among others [5]. Akin to social stereotypes, these biases are omnipresent in language’s statistical structure and, as such, they are encoded in LLMs [6]. For example, language statistics carry information about the way we imagine geographical maps, or about how we perceive differences between numbers and the quantities they represent [7]. Similarly, word co-occurrence dynamics reflect our decision-making and higher-order reasoning processes: different patterns of word co-occurrence form different sentences, each carrying its particular meaning. Indeed, how a sentence or question is worded matters, and some questions are more consequential than others. For instance, upon being told that there exist two treatments for a disease (treatment A has a 10% mortality rate, while treatment B has a 90% survival rate), most people will be inclined to choose treatment B because it is framed more positively, i.e., in terms of survival rather than death – notwithstanding the fact that the two statements are mathematically identical. Such framing bias [5] –namely the fact that our choices are guided by how scenarios are framed, here translating into an aversion to loss– has been found in LLMs, alongside several others [6] [Fig. 1B].

As shown, cognitive schemas translate into specific linguistic patterns. Since LLMs can exhibit patterns that resemble human heuristics, it is intuitive to expect that increased LLMs’ usage will contribute to the spreading, reinforcement and consequent amplification of such heuristics. As a result, efforts to characterize and mitigate cognitive biases in LLMs have promptly initiated [8]. However, while broad consensus exists over the fate of social stereotypes (i.e., inasmuch as possible, they are to be lessened), a discussion on how to behave with respect to cognitive biases has not been undertaken. If cognitive biases are inherent to how humans cognize (and thus unlikely to be acquired through exposure), why are scholars mitigating them? In other terms: Is cognitive bias mitigation actually desirable?

### Assumptions Made Explicit

As anticipated, cognitive bias mitigation is prima facie unproblematic [8]. On a closer look, however, it rests on two underlying premises which –although often taken for granted in the specific context of cognitive bias mitigation– are the subject of substantial scientific debate. Engaging with these assumptions, which we outline below, casts doubt on the suitability of mitigation efforts.

First, is the idea that cognitive biases do not benefit cognition (whilst their mitigation does). Indeed, cognitive biases often determine errors and deviations from rational (i.e., consistent and coherent [5]) thinking, viz. our cognitive schemas do not always lead to accurate depictions of reality. For example, our geographical mental maps do not necessarily reflect factual, physical maps, nor do our intuitions about numbers accurately reflect mathematically sound number lines [7]. Were the algorithmic representations learned by LLMs cleansed of fallacious heuristics, our cognitive processes would upgrade to being more objective, consistent, and ultimately free of error.

Deciding that cognitive biases should be mitigated en masse, however, is not immediately obvious. Historically, cognitive biases are associated to a series of misconceptions [9], such as the belief that they are second-best to logical reasoning, or that they arise from cognitive limitations. Notwithstanding their somewhat negative framing, however, these biases are a fundamental part of human cognition, which allows us to reach satisfactory solutions across countless situations. Take the aforementioned framing bias [5]. The fact that humans more willingly accept treatment B over treatment A opposes logic but, at the same time, logic is not all that determines our attitude towards an event: our knowledge, beliefs and contingent life circumstances also play a role. For instance, physicians might frame a given treatment in a positive vs. negative way considering the alternatives that are offered by modern medicine, providing patients an anchor to hold onto while making a decision. In short, a cognitive bias can be either conceptualized as an error, or as a context-specific adaptation. While cognitive bias mitigation has initiated, with more recent models displaying less biases of this sort [10], the implications of such practice for cognition remain to date largely unaddressed.

Second, there is the conjecture that mitigating cognitive biases yields rational, objective, and ultimately neutral decision-making LLM technologies. However, in this context cognitive bias-free is arguably not synonymous with neutral, as acting on a cognitive bias presupposes a normative choice as per how such bias should be corrected. This is especially evident in decision-making conundrums where, upon choosing between mutually-exclusive options, none of them can ever be inherently neutral. In a trolley-like scenario, for instance, a self-driving car is headed to crush towards a group of pedestrians. By turning left, only one pedestrian is hit instead of three. A self-driving car subject to the inaction bias will not change its trajectory to save the group of pedestrians, with this option not being a neutral stance. Thus, from a logical standpoint, cognitive bias-free LLMs cannot be regarded as intrinsically preferable decision makers to their bias-replenished counterparts.

To an extent, cognitive biases are perceived as if they dirtied neutral, coherent thinking. This view, which is reminiscent of philosophical traditions which privilege universality and objectivity over the complex and dynamic nature of reality [11], is also the one implicitly adopted upon attempting bias mitigation. The assumption is that, underneath brittle heuristics, lie ground-truth knowledge and infallible reasoning strategies. However, we know that algorithms are not neutral data processors.

Deployed in the real world, the non-neutral nature of AI technologies requires scholarly and societal attention, especially as humans tend to excessively trust automated tools (the so-called automation bias [3]). LLMs are often anthropomorphized as intelligent agents capable of better-than-human reasoning [12], whose choices are supposedly impartial, fair and driven by mathematically rigorous analyses. In addition, generative AI technologies display confident attitudes that make their outputs go largely unchallenged. To wit, even if what a model says is wrong, humans tend to believe it [12]. From the above considerations, it follows that not only bias-free neutrality in LLMs might be unachievable, but also that, in and of itself, the bias-correction process towards such goal fosters the wrong idea that what is bias-free is also neutral. This reasoning applies to both cognitive biases that resemble the biases of humans, and to those biases that have been found in LLMs only (e.g., the “yes response bias” in [13] and the “yes-no bias” in [14]).

### The Ethics of Intervening

Taken together, the above-outlined assumptions –namely that cognitive biases do not benefit cognition, and that their mitigation obtains objective outputs– raise the question of whether mitigation efforts should be pursued at all. For the sake of argument, however, let’s assume that cognitive bias mitigation is uncontroversially desirable. At this stage, the process of intervening on cognitive biases demands a decision as per how to intervene. We rather argue that cognitive bias mitigation itself introduces an additional and nontrivial ethical challenge, namely determining which cognitive biases (if any), we want LLMs to encode.

From an ethical standpoint, cognitive biases are as relevant as social ones. However, a big difference between the two is that while a social bias can be helped by balancing out datasets such that unbiased algorithmic representations are obtained, cognitive biases are harder to correct, in that they are pervasive in language and presuppose a choice as per how they should be fixed. Humans routinely rely on cognitive biases and heuristics to guide our decisions and, as anticipated, such reliance is successful in many –but crucially not all– circumstances. Thus, an intuitive criterion to determine whether to preserve a cognitive bias in LLMs is based on the extent to which a given bias is adaptive and functional. Concisely, what to make of a bias could be decided upon how well it performs in the world. This possibility, in turn, brings about the aforesaid challenge: because the functionality of a bias varies across contexts, it cannot be universally determined.

The deployment of LLMs is nowadays conceived at a nearly-global scale. However, various –albeit not all– human cognitive preferences vary based on circumstances that play out in much smaller dimensions, such as membership in a particular age, clinical, linguistic or cultural group, in addition to motivational and individual inclinations. Plus, while LLMs’ reasoning is only partially sensitive to situational factors, human reasoning is ineluctably tied to context. For instance, the framing bias entails that scenarios which have identical outcomes lead to different choices as a function of how the initial scenarios are presented [5]; this means that different humans will reach different conclusions based on different heuristics and situations.

This matter carries important ethical implications. Indeed, judging the functionality of a bias presupposes a choice over which outcome (out of a set of possible outcomes) is most desirable. For instance, Cheung et al. [14] investigated moral decision-making in LLMs, showing that the evaluation of a model’s moral advice depends on many factors: moral, ethical, and juridical [14], among others. In essence, by adjusting the degrees of presence (or absence) of a cognitive bias, LLMs amplify selected cognizing and decision-making products which are not universally shared, and thus representative of picked-out worldviews. Ultimately, while characterizing the cognitive biases at play in LLMs at a descriptive level is an uncontroversial practice, normative work calls for nuanced criticism.

### Prospects

In this Comment, we raised the question of whether cognitive bias mitigation in LLMs is desirable. In attempting to identify the motivations behind such efforts, we hypothesized that they are fostered by two assumptions: (i) the idea that these biases are a drawback to consistent, coherent reasoning, and (ii) the conjecture that bias-mitigated technology obtains neutral outputs, surpassing its biased equivalent. We have argued that these are two questionable assumptions, raising doubts about the appropriateness of mitigation efforts. Subsequently, even speculating that cognitive bias mitigation is uncontroversial, we have shown that mitigation itself introduces a further, nontrivial ethical challenge: choosing which cognitive biases should, or should not, be encoded in LLMs. While cognitive bias mitigation is ongoing, to date these three subjects are yet to be collectively discussed with reference to AI.

So, how should we proceed? Two options can be envisaged. The first is to attempt mitigating cognitive biases en bloc, a strategy based on the view that human-like biases in LLMs are obstacles to developing consistent reasoning and reliable decision-making in these systems [8]. This approach sets the functional role of cognitive biases to the side and embraces the possibility of wholly logical thinking as a desirable standard. Alternatively, cognitive biases could be retained based on their functionality, i.e., maintained only if they perform well in real-world contexts. This approach demands the integration of domain-specific and ethical perspectives: evaluating functionality entails deciding what counts as performing well, a judgment that is inherently circumstantial and reflects interests and experiences of a subset of the population.

Regardless of the direction the scientific community chooses to pursue, it is pivotal that we are clear on what the end goal of cognitive bias mitigation is (as is the case with social biases), such that the processes of (i) sifting through cognitive biases to pick the ones we wish to exclude, (ii) developing methods that work towards their mitigation, and (iii) measuring the effectiveness of our efforts, can take place on a sound epistemological basis [15]. Indeed, whilst different, both scenarios described above implicitly entail one questionable premise: the belief that it is possible to build an AI that is objective, raw and purged of (non-functional) human conceptualizing. According to this view, by cleansing LLMs of heuristics and biases of cognition, we will finally reach an unmediated grasp of reality.

## Standfirst

Large Language Models include not only social stereotypes, but cognitive biases. As efforts to detect, characterize and rectify cognitive biases in LLMs have started, we urge the scientific community to engage in a critical discussion: cognitive biases are often conceptualized as errors, but they also reflect functional, context-specific reasoning adaptations.
